# To treat or not to treat: metabolomics reveals biomarkers for treatment indication in chronic lymphocytic leukaemia patients

**DOI:** 10.18632/oncotarget.8078

**Published:** 2016-03-14

**Authors:** Jaroslaw Piszcz, Emily G. Armitage, Alessia Ferrarini, Francisco J. Rupérez, Agnieszka Kulczynska, Lukasz Bolkun, Janusz Kloczko, Adam Kretowski, Alina Urbanowicz, Michal Ciborowski, Coral Barbas

**Affiliations:** ^1^ Department of Haematology, Medical University of Bialystok, Bialystok, Poland; ^2^ CEMBIO, Centre for Metabolomics and Bioanalysis, San Pablo CEU University, Madrid, Spain; ^3^ Clinical Research Centre, Medical University of Bialystok, Bialystok, Poland; ^4^ Clinical Oncology and Hematology Department, Provincial Hospital, Suwalki, Poland

**Keywords:** chronic lymphocytic leukaemia, metabolomics, disease staging, biomarker, acylcarnitines

## Abstract

In chronic lymphocytic leukaemia (CLL), the clinical course of patients is heterogeneous. Some present an aggressive disease onset and require immediate therapy, while others remain without treatment for years. Current disease staging systems developed by Rai and Binet may be useful in forecasting patient survival time, but do not discriminate between stable and progressive forms of the disease in the early stages. Recently ample attention has been directed towards identifying new disease prognostic markers capable of predicting clinical aggressiveness at diagnosis. In the present study serum samples from stable (*n* = 51) and progressive (*n* = 42) CLL patients and controls (*n* = 45) were used with aim to discover metabolic indicators of disease status. First an LC-MS based metabolic fingerprinting method was used to analyse selected samples in order to find a potential markers discriminating aggressive from indolent patients. Ten of these discovered markers were validated on the whole set of samples with an independent analytical technique. Linoleamide (*p* = 0.002) in addition to various acylcarnitines (*p* = 0.001–0.000001) showed to be significant markers of CLL in its aggressive form. Acetylcarnitine (*p* = 0.05) and hexannoylcarnitine (*p* = 0.005) were also distinguishable markers of indolent subjects. Forming a panel of selected acylcarnitines and fatty acid amides, it was possible to reach a potentially highly specific and sensitive diagnostic approach (AUC = 0.766).

## INTRODUCTION

Chronic lymphocytic leukaemia (CLL) is one of the most common lymphoid malignancies. Although a large majority of patients are diagnosed in the early stages of the disease, their clinical course is heterogeneous. Some patients present with an aggressive disease onset and require immediate cytostatic therapy, while others remain without treatment for many years [1]. Disease staging systems developed by Rai [2] and Binet [3], which divide patients into good, intermediate and poor prognostic groups, may be useful in forecasting patient survival time. Unfortunately, the systems do not discriminate between stable and progressive forms of the disease in the early stages [4]. Over the last decade, ample attention has been directed towards identifying new disease prognostic markers capable of predicting clinical aggressiveness at diagnosis. The mutational status of the immunoglobulin heavy chain variable region (IGHV) gene has been proved to be one of such markers. Patients carrying un-mutated IGHV (UM-IGHV) genes show poor prognosis, reduced survival rates and response to chemotherapy [5]. Another examination that corresponds well with disease prognosis and may be used as an alternative marker for IGHV mutational status is the assessment of HO(ZAP70) and CD38. Leukemia-cell expression of ZAP-70 or CD38 has been found to correlate with the expression of un-mutated IGHV genes [6, 7]; yet the association between the former and the latter is not absolute. Another powerful prognostic is the presence of cytogenetic lesions. The most common deletions concern the long arm of chromosome 13 [del(13q14.1)], the trisomy of chromosome 12, deletions in the long arm of chromosomes 11 [del(11q)] or 6 [del(6q)] and in the short arm of chromosome 17 [del(17p)] [8]. There is increasing evidence from prospective clinical trials that detection of del(17p) in CLL patients heralds an inferior prognosis and resistance to standard chemotherapy regimens using alkylating drugs and/or purine analogs [9, 10].

Although the aforementioned prognostic markers correlate to a large extent with the disease course, they do not determine treatment indication *per se*. Current criteria for initiating CLL treatment are rather based on clinical features [11]. For instance, it is established in general practice that newly diagnosed patients with asymptomatic early-stage disease (Rai O, Binet A) should be monitored without therapy unless there is evidence of disease progression. Whereas patients at intermediate (stages I and II) and high risk (stages III and IV) stages (according to the modified Rai classification), or at stage B or C (following the Binet system) usually benefit from the initiation of treatment. Some of them (in particular Rai intermediate risk or Binet stage B) can be monitored without therapy until evidence of progressive or symptomatic disease is found. Active disease is recognized when at least one of the criteria presented in Table S1 (found in Supplementary Information) is met.

Regardless of the presence or absence of poor prognostic factors, CLL treatment starts once the symptoms of disease progression have occurred. The available evidence indicates that treatment of unselected early-stage patients with alkylating agents promptly after diagnosis offers no survival advantage over treatment commenced at the time of disease progression [4].

Changes of inner leukemic cell function or its interactions with other immune and micro-environmental cells may contribute to rendering the disease process aggressive [12]. Certain alterations in metabolites and small molecules involved in biochemical processes provide a functional reflection of a possible pathology. Metabolic fingerprinting techniques (for instance, gas/liquid chromatography mass spectrometry (MS), nuclear magnetic resonance (NMR)) combined with multivariate statistics (interchangeably termed ‘metabolomics’ or ‘metabonomics’) offer the ability to examine global changes in metabolites associated with physiological conditions [13]. Thus, metabolomics is a powerful approach for examining disease-related metabolic changes and, accordingly, proves highly effective in identifying new biomarkers [14]. Several studies have highlighted the potential of metabolomics as a tool for cancer detection, progression or assessment of treatment effect. Capabilities and current discoveries of metabolomics tools in cancer research have been recently reviewed [15, 16].

Until now, a metabolomics approach has been applied in only a few studies to investigate chronic lymphocytic leukaemia [17–19]. In one of these studies 1H-NMR was employed to detect plasma metabolites and showed different metabolic profiles of early-stage, untreated CLL patients (Binet stage A) according to IGHV mutational status and ZAP70 [17]. In the other study, MS based metabolomics was used to find differences between CLL cells obtained from patients in aggressive and indolent states [19]. In this research, we focus on the assessment of metabolic changes in serum of aggressive and indolent CLL patients by means of liquid chromatography – mass spectrometry (LC-MS). Our methodology has been used to discriminate between serum fingerprints of B-cell non-Hodgkin's lymphomas recently [18]. We have extended on this in the study presented by first applying our methodology to find differences in serum fingerprints of both aggressive and indolent CLL patients relative to controls with the purpose of elucidating metabolic fingerprints, which may be useful as indicators of disease status. Furthermore, we have performed validation on a panel of markers, identifying metabolites that have the highest specificity and selectivity for discriminating CLL from controls and moreover indolent and aggressive states of CLL. Serum was selected as the sample of choice due to its utility and applicability in diagnostics. Application of the LC-MS technology for biomarker discovery is not only complementary to NMR [20], but also boasts several advantages over it, including greater sensitivity and ability to measure large number of both high and low-intense metabolites. Obtained results may help in classification of patient status and may reflect leukaemia progression in newly diagnosed patients.

## RESULTS

### Quality control of the methodologies

**Figure 1:**
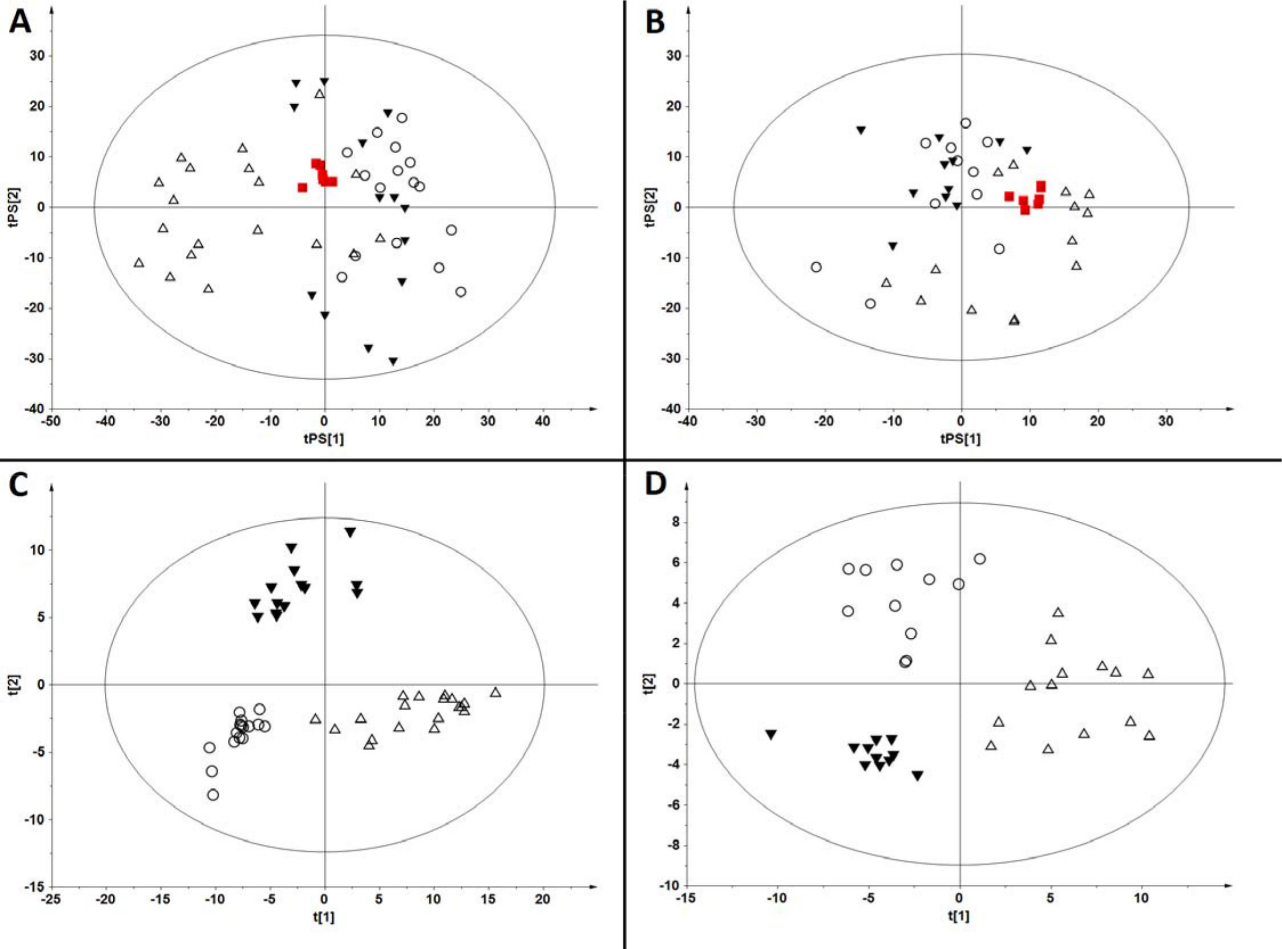
Multivariate analyses of samples analysed by fingerprinting method in positive and negative ESI modes (**A**) PCA model (R2 = 0.137) built for samples analyzed in ESI (+) mode with prediction of QC samples. (**B**) PCA model (R2 = 0.331) built for samples analyzed in ESI (−) mode with prediction of QC samples. Variables after QA protocol were used to build models from panels (A) and (B). (**C**) Classification of samples on PLS-DA model (R2 = 0.954, Q2 = 0.737) built for filtered data generated in ESI (+) mode. (**D**) Classification of samples on PLS-DA model (R2 = 0.985, Q2 = 0.662) built for filtered data generated in ESI (−) mode. ■ - QC, △ - control, ▼ - indolent, ○ - aggressive.

Quality checking for fingerprinting data was performed separately for positive and negative ion modes. In both cases chromatograms obtained for participants serum samples and for quality control samples (QCs) were aligned together and filtered to obtain features present in > 50% of QCs and with relative standard deviations (RSDs) < 30% in QCs yielding datasets with 3170 features for ESI+ and 1253 for ESI-mode. Principal components analysis (PCA) was used to provide an overview of the obtained data sets after Pareto-scaling. Close clustering of QC samples was observed in ESI+ (Figure 1A) and ESI- mode (Figure 1B) reflecting the system's stability and performance, as well as the reproducibility of the sample treatment procedure.

Quality of QQQ analyses was checked by calculation of RSD for metabolites in QC samples, which was between 3 and 17%.

### Results of fingerprinting study

In order to perform sample classification, the dataset obtained after filtering of the QCs was additionally filtered by choosing only metabolic features present in 100% of the samples in at least one of the groups (C, A, I). For these data sets (1175 features in ESI+, and 571 in ESI- mode) partial least squares discriminant analysis (PLS-DA) models were built showing clear classification of samples in ESI+ (Figure 1C) as well as in ESI- (Figure 1D) mode. The quality of the models was excellent with variance explained (R2 = 0.954) and variance predicted (Q2 = 0.737) for ESI+, and R2 = 0.985, Q2 = 0.662 for ESI- mode. Obtained models were validated by cross-validation using the leave 1/3 out approach [21]. In the models obtained for data recorded in ESI+ mode, all excluded samples were classified correctly in 94 ± 7%, while in the models obtained for data recorded in ESI- mode the percentage of samples classified correctly was calculated as 83 ± 7%.

Statistical analysis of fingerprinting data was conducted as described in the materials and methods section. Statistical analysis (*p* ≤ 0.05) was performed for the comparisons of C *vs* A, C *vs* I, and A *vs* I in each ionisation mode giving 284, 184 and 125 features in ESI+ and 147, 139, and 15 features in ESI- ionisation modes respectively. Identification of significant metabolites was confirmed by MS/MS fragmentation or analysis of the standards. Identified metabolites are summarised in Tables 1–3 including retention time, theoretical mass and error of measured mass, MS/MS fragments and the percentage of change between the groups in the comparisons performed.

**Table 1:** Identification of lysophospholipids that were significantly differentiating plasma profiles of CLL patients from controls

Compound	RT (min)	Theoretical mass (Da)	Mass error (ppm)	Identification	Change [%] (*p*-value)		
					I vs C	A vs C	A vs I
Lyso PC (16:0)	20.1	481.3532	−2.1	P: 184.072, 104.107, 86.096	−32 (0.0005*)	−35 (0.00004*)	NS
Lyso PC (16:1)	17.4	493.3168	−3.2	P: 476.309, 184.071, 104.107, 86.096	−26 (0.008*)	−28 (0.005*)	NS
Lyso PC (17:0)	21.3	509.3481	0.2	P: 492.341, 184.071, 104.107, 86.096	NS	−23 (0.004*)	NS
Lyso PC (18:0)	24.5	509.3845	−2.6	P: 184.073, 104.107, 86.097	−32 (0.003*)	−34 (0.0003*)	NS
Lyso PC (18:1)	21.0	507.3689	−1.4	P: 184.072, 104.107, 86.096	−34 (0.0002*)	−33 (0.0002*)	NS
Lyso PC (18:2)	17.7	519.3325	3.3	P: 502.327, 184.072, 104.107, 86.096	−19 (0.04)	−19 (0.03)	NS
Lyso PC (20:0)	25.1	535.4001	−3.5	P: 184.071, 104.107, 86.096	−45 (0.000005*)	−38 (0.001*)	NS
Lyso PC (20:1)	24.0	549.3794	−2.2	P: 184.072, 104.107, 86.096	NS	−25 (0.009*)	NS
Lyso PC (20:4)	17.7	543.3325	1.3	P: 526.329, 184.073, 104.107, 86.097	−21 (0.05)	−17 (0.08)	NS
PC (17:0/2:0)	21.1	551.3587	−5.3	N: 492.345, 269.248, 224.069, 78.96	−61 (0.00000009*)	−53 (0.000001*)	NS
Lyso PE (O-16:0)	19.8	439.3063	−3.4	N: 377.241, 196.036, 140.011, 78.959	−42 (0.02)	−41 (0.03*)	NS
Lyso PE (16:0)	20.0	437.2906	−4.1	N: 239.235, 196.036, 140.009, 78.959	−45 (0.0002*)	−51 (0.0001*)	NS
Lyso PE (18:1)	24.3	465.3219	−2.4	N: 403.26, 267.267, 196.037, 140.011, 78.959	−43 (0.001*)	−46 (0.0009*)	NS
Lyso PE (20:0)	28.8	493.3532	−2.8	N: 295.297, 196.038, 140.013, 78.959	−48 (0.0001*)	−45 (0.0003*)	NS
Lyso PE (20:3)	19.0	503.3012	−2.0	P: 363.289	+ 39 (0.02)	NS	−25 (0.05)
Lyso PA (20:4)	24.3	458.2433	1.1	N: 303.233, 259.242, 171.006, 152.996, 96.969, 78.959	−67 (0.002*)	−63 (0.00006*)	NS
Lyso PI (16:0)	23.0	572.2962	−2.4	N: 391.224, 315.048, 255.232, 241.011, 152.996, 78.959	−46 (0.002*)	−52 (0.0002*)	NS
Lyso PI (18:1)	25.0	598.3118	−3.3	N: 417.239, 315.048, 281.247, 241.011, 152.995	−31 (0.04)	−32 (0.02)	NS
Lyso PI (18:2)	20.6	596.2961	−3.9	N: 415.222, 279.231, 241.01, 152.995, 78.959	−37 (0.008*)	−38 (0.0004*)	NS

P, N – metabolite identified in positive or negative ESI mode, respectively; NS – non-significant; * - these *p*-values remain < 0.05 after correction by FDR; N vs C - (+)/(−) means increased/decreased abundance in CLL patients who do not require treatment in comparison to controls; A vs C - (+)/(−) means increased/decreased abundance in CLL patients in aggressive state of the disease in comparison to controls; A vs I - (+)/(−) means increased/decreased abundance in CLL patients in aggressive state as compared CLL patients in indolent state of the disease.

**Table 2:** Identification of fatty acid amides, sphingolipids and fatty acids significantly differentiating plasma profiles of CLL patients from controls

Compound	RT (min)	Theoretical mass (Da)	Mass error (ppm)	Identification	Change [%] (*p*-value)		
					I vs C	A vs C	A vs I
dodecanamide^S^	16.8	199.1936	−2.0	P: 116.104, 102.09, 88.076	−NS	+ 64 (0.05)	+ 87 (0.01*)
Linoleamide^S^	24.8	279.2562	−3.6	P: 263.235, 245.224, 175.146, 161.132, 133.098, 109.1, 95.085, 81.07, 69.07, 57.07, 43.055	NS	+ 90 (0.0008*)	+ 83 (0.05)
Oleamide^S^	28.0	281.2719	1.1	P: 265.251, 247.24, 177.162, 163.146, 149.131, 135.116, 97.101, 83.086, 69.07, 57.071	NS	+ 28 (0.05)	NS
Palmitoylethanolamide	25.2	299.2824	−4.0	P: 283.262, 62.061, 44.051	+ 18 (0.01)	NS	NS
Hydroxysphingosine	13.5	315.2773	−6.7	P: 106.086, 88.075, 57.071	−32 (0.003*)	−39 (0.002*)	NS
Sphingosine-1-phosphate	14.9	379.2488	−1.6	P: 264.267, 82.065	−40 (0.000007*)	−35 (0.0001*)	NS
			−2.6	N: 78.959	−52 (0.00008*)	−37 (0.01*)	NS
Sphinganine-phosphate	15.8	381.2644	−0.5	P: 364.247, 284.293, 266.28	−47 (0.00002*)	−37 (0.001*)	NS
			−4.2	N: 78.959	−43 (0.00007*)	−40 (0.002*)	NS
Leukotriene B4	14.2	336.2301	−3.9	N: 335.221, 317.211, 195.101, 129.054, 71.014, 59.014	Not detected in CLL samples (< 0.000001*)	Not detected in CLL samples (< 0.000001*)	−
hydroxy-eicosatetraenoic acid	20.3	320.2285	7.2	N: 319.223, 301.214, 275.233, 257.225, 179.106, 163.11, 135.116, 59.014	−81 (0.0001*)	−74 (0.0001*)	NS
Eicosapentaenoic acid (dehydroarachidonic)	25.7	302.2246	−2.6	N: 301.216, 283.208, 257.227, 229.194, 203.179, 177.092, 59.014	NS	−59 (0.0004*)	−54 (0.03)
Eicosatetraenoic acid	28.1	304.2402	−4.6	N: 303.231, 285.22, 259.242, 59.015	−47 (0.0002*)	−55 (0.00001*)	NS
Docosapentaenoic acid	28.7	330.2559	−3.3	N: 285.255, 59.014	+ 50 (0.04)	NS	−33 (0.05)
Palmitic acid^S^	31.3	256.2402	0.0	P: 239.118, 212.234, 135.117, 117.091, 103.075, 89.06, 71.086, 57.071, 43.056	+ 17 (0.06)	+ 28 (0.02)	NS

S – Identity of these metabolites was confirmed by the LC-MS/MS analysis of the standards; P, N – metabolite identified in positive or negative ESI mode, respectively; NS – non-significant; * - these *p*-values remain < 0.05 after correction by FDR; I vs C - (+)/(−) means increased/decreased abundance in CLL patients in indolent state of the disease in comparison to controls; A vs C - (+)/(−) means increased/decreased abundance in CLL patients in aggressive state of the disease as compared to controls; A vs I - (+)/(−) means increased/decreased abundance in CLL patients in aggressive state in comparison to CLL patients in indolent of the disease.

**Table 3:** Identification of other metabolites significantly differentiating plasma profiles of CLL patients from controls

Compound	RT (min)	Monoisotopic mass (Da)	Mass error (ppm)	Identification	Change [%] (*p*-value)		
					I vs C	A vs C	A vs I
Acetylcarnitine^S^	0.7	203.1158	−2.9	P: 145.048, 85.028, 60.081	+ 36 (0.02*)	+ 52 (0.0008*)	NS
Hexanoylcarnitine^S^	1.1	259.1783	−4.2	P: 201.111, 85.028, 60.08	NS	+ 107 (0.02)	+ 102 (0.04)
Oxo-methylthioheptanoic acid	0.6	190.0663	4.7	P: 173.027	NS	NS	−26 (0.05)
ornithine^S^	0.6	132.0899	−0.8	N: 86.977, 44.999	NS	−26 (0.02)	NS
piperidine	0.9	85.0891	−10.6	P: 69.07, 44.05, 43.055, 41.04, 30.035	−22 (0.04)	−28 (0.04)	NS
Phe Phe	1.0	312.1474	−3.8	P: 166.085, 120.08	NS	−39 (0.006*)	NS
			−1.9	N: 250.122, 175.087, 164.071, 147.044, 91.055, 71.026	NS	−31 (0.04)	NS
phenylacetylglutamine	1.0	264.111	−4.5	N: 145.061, 127.05, 109.039	NS	−45 (0.01*)	NS
			0.8	P: 130.051, 147.074	−28 (0.06)	−49 (0.001*)	NS
Cresol sulfate	1.7	188.0143	−2.7	N: 107.05, 79.958	NS	−60 (0.007*)	NS
Propionaldehyde	4.2	58.0419	9.1	P: 43.019, 31.019	NS	−18 (0.03)	NS
Unsaturated hydroxy (or oxo) fatty aldehyde OR Hexynoic/hexadienoic acid	4.2	112.0524	−1.8	P: 55.019	NS	−16 (0.03)	NS
	9.6	112.0524	−1.8	P: 55.018	NS	−31 (0.004*)	NS
Biliverdin	10.3	582.2478	−2.7	P: 297.123	−40 (0.08)	−47 (0.03)	NS
hexadecatrienol	23.3	236.214	−3.4	P: 219.209, 149.129, 135.117, 109.101, 95.085, 83.085, 57.07	NS	+ 32 (0.05)	NS
hydroxy-phosphonooxy-octadecanoic acid	27.5	396.2277	−2.3	N: 327.232, 283.242, 44.999	NS	NS	−35 (0.03)
Octadecatrienol	28.0	264.2453	−0.4	P: 247.24, 163.146, 149.13, 135.116, 121.1, 109.1, 95.085, 81.07, 69.07, 57.071	NS	+ 27 (0.03)	NS

S – Identity of these metabolites was confirmed by the LC-MS/MS analysis of the standards; P, N – metabolite identified in positive or negative ESI mode, respectively; NS – non-significant; * - these *p*-values remain < 0.05 after correction by FDR; I vs C - (+)/(−) means increased/decreased abundance in CLL patients in indolent state of the disease in comparison to controls; A vs C - (+)/(−) means increased/decreased abundance in CLL patients in aggressive state of the disease as compared to controls; A vs I - (+)/(−) means increased/decreased abundance in CLL patients in aggressive state in comparison to CLL patients in indolent of the disease.

### Results of validation study

**Figure 2:**
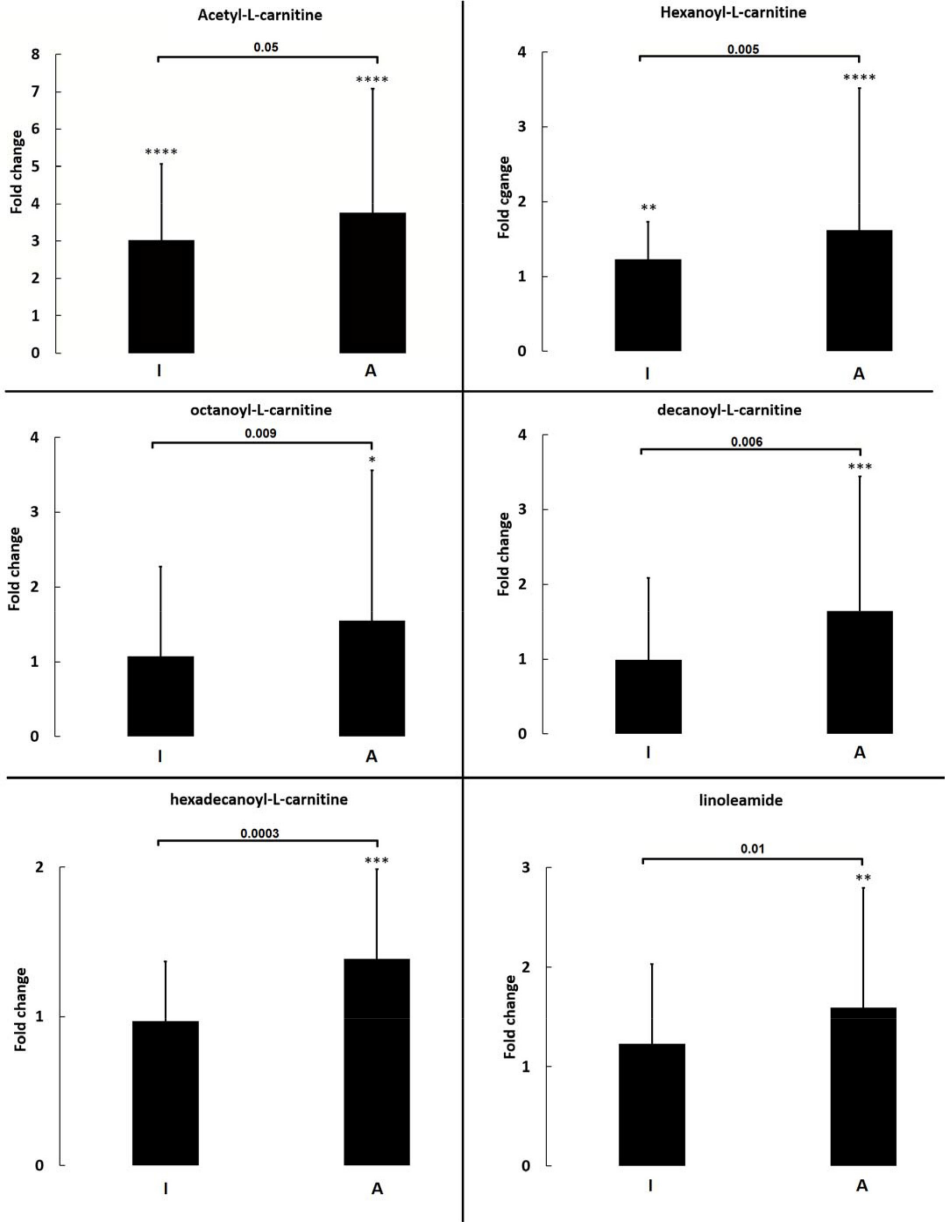
Fold change of selected metabolites quantified in validation study Each bar represents median of fold change with inter quartile range for metabolites as calculated for indolent (I) or aggressive (A) CLL patients in comparison to controls. Significant differences between I and A are indicated by *p*-value, while between controls and A or I by asterisks. *p ≤ 0.001, **p ≤ 0.0001, ***p ≤ 0.00001, ****p ≤ 0.000001, no indication means not significant.

Out of ten metabolites selected for validation, six (Figure 2) were found significantly higher in the aggressive state of the disease compared to indolent or controls. These metabolites were acylcarnitines (acetyl, hexanoyl, octanoyl, decanoyl, hexadecanoyl) and linoleamide. Among them acetylcarnitine and hexanoylcarnitine can be used to discriminate between controls and indolent patients. To evaluate the clinical utility of these metabolites as potential biomarkers to diagnose CLL, ROC curves were used. ROC analyses were performed to check the utility of metabolites selected for validation (individually or grouped) to classify patients as having CLL in general or as having an aggressive state of the disease (Table 4). As it can be seen in Table 4, metabolites best classifying patients as having CLL are acetylcarinite, hexanoylcarnitine and octanoylcarnitine. Obtained results of area under the curve (AUC), sensitivity, and specificity slightly improved when these acylcarnitines were grouped. Similarly, ROC analyses indicate that acylcarnitines are promising biomarkers of CLL status; however better results were obtained for combination of selected acylcarnitines and fatty acid amides (Table 4).

**Table 4:** ROC analysis to evaluate utility of validated metabolites as biomarkers of CLL in general and in its aggressive state

Utility of validated metabolites as biomarkers of CLL					
Metabolite	AUC	Sensitivity (%)	Specificity (%)	PPV (%)	NPV (%)
Acetylcarnitine	0.773	97.8	48.9	65.7	95.7
Butyrylcarnitine	0.599	100	0	50.0	0.0
Hexanoylcarnitine	0.697	88.0	42.2	60.4	77.9
Octanoylcarnitine	0.616	85.9	31.1	55.5	68.8
Decanoylcarnitine	0.623	95.6	11.1	51.8	71.6
Palmitoylcarnitine	0.57	100	2.2	50.6	100.0
Dodecanamide	0.324	100	0	50.0	0.0
Hexadecanamide	0.397	98.9	2.2	50.3	66.7
Oleamide	0.492	98.9	2.2	50.3	66.7
Linoleamide	0.601	100	0	50.0	0.0
Acetylcarnitine Hexanoylcarnitine Octanoylcarnitine	0.769	97.8	53.3	67.7	96.0
Acetylcarnitine Hexanoylcarnitine	0.766	96.7	51.2	66.5	93.9
**Utility of validated metabolites as biomarkers of aggressive state of CLL**					
Acetylcarnitine	0.695	43.2	93.0	86.1	62.1
Butyrylcarnitine	0.548	10.8	98.0	84.4	52.4
Hexanoylcarnitine	0.690	27.0	96.0	87.1	56.8
Octanoylcarnitine	0.651	29.7	95.0	85.6	57.5
Decanoylcarnitine	0.662	27.0	94.0	81.8	56.3
Palmitoylcarnitine	0.719	40.5	94.0	87.1	61.2
Dodecanamide	0.497	8.1	100.0	100.0	52.1
Hexadecanamide	0.516	5.4	100.0	100.0	51.4
Oleamide	0.600	18.9	96.0	82.5	54.2
Linoleamide	0.672	16.2	98.0	89.0	53.9
Acylcarnitines^a^	0.743	32.4	95.0	86.6	58.4
FAA^b^	0.662	13.9	96.0	77.6	52.7
Acylcarnitines and FAA	0.750	54.0	89.0	83.1	65.9

ROC - receiver operating characteristic; AUC - area under the curve; PPV - positive predictive value; NPV - negative predictive value, FAA – fatty acid amides.

a Following acylcarnitines were grouped for ROC analysis: acetyl-, hexanoyl-, octanoyl-, decanoyl- and palmitoyl-.

b Following fatty acid amides were grouped for ROC analysis: oleamide and linoleamide.

## DISCUSSION

CLL, one of the most common adult blood cancer in the western world [22] is described as exhibiting heterogeneous clinical behaviour such that survival can range from months to decades and that the disease can be presented in aggressive (requiring immediate treatment) and indolent forms [17]. Despite a plethora of risk factors, CLL therapy initiation is still based on clinical features and currently the diagnosis of CLL does not always lead to prompt treatment due to potentially harmful consequences for the patient. Metabolomics and lipidomics strategies have been previously applied to study CLL [18, 19]. However we present, to the best of our knowledge, the first comparison of serum fingerprints from patients that possess either the indolent or aggressive form of CLL as well as serum from healthy volunteers in order to observe the differences caused by the disease, that have led to the proposal of a panel of biomarkers with the required sensitivity and specificity to improve diagnosis, prognosis and proposed treatment of the disease. The significant differences identified from this global examination of serum-derived metabolites in the context of CLL are highlighted in Tables 1–3.

In a previous study comparing different B-cell malignancies, we revealed characteristic metabolites of CLL that followed the same trends as in the present study with respect to the disease versus controls and additionally, here, with respect to aggressive subjects compared to indolents. These include acetylcarnitine, leukotriene B4 (LTB4), eicosapentaenoic acid (dehydroarachidonic) acid, eicosatetraenoic acid, sphingosine-1-phosphate and phenylacetylglutamine in addition to some (lyso) phospholipids [18].

One particularly interesting marker revealed for the first time in the present study that was able to significantly discriminate all groups from each other was biliverdin. Its concentration (that changed with a remarkable percentage between groups) is likely to be inversely related to aggressiveness of CLL. Human biliverdin reductase has been described as a cytoprotectant and its expression in multidrug resistant leukaemic HL60 cells has been reported to be significantly increased [23]. Expression of haem oxygenase-1 (HO-1) is also found to be increased in cancer cells and is further enhanced following chemotherapeutic treatment [24]. It provides protection against cellular stress and converts haem into carbon monoxide, free iron and biliverdin, the latter of which that is later converted to potent antioxidant bilirubin [25]. Moreover, in a study of acute myeloid leukemia, it has been reported that silencing HO-1 significantly increases *in vitro* chemosensitivity [26]. Through one or both of these mechanisms, our data suggest that biliverdin is rapidly reduced in CLL that is dependent on the aggressiveness of the disease.

Other key metabolites were fatty acids. Among them, two saturated fatty acids (myristic and palmitic acids) were found significantly higher in aggressive CLL than in controls. Regarding polyunsaturated fatty acids (PUFAs) hydroxy-eicosatetraenoic, Eicosapentaenoic, and Eicosatetraenoic were significantly lower in CLL relative to controls and moreover lower in aggressive relative to indolent patient samples. Another PUFA - docosapentaenoic acid was found showing paradoxical results, because it was not significantly different between controls and aggressive CLL, but it was higher in indolents as compared to both to aggressive and CLL. Despite the fact that more data are needed to confirm this trend, the differences found in the present study in the metabolism of PUFA deserve further attention, because several cancers, including CLL have been previously associated with activating the oxidative cascade of PUFAs [22]. Fatty acids and their involvement in leukaemia have received much attention in the literature. For example it was recently proposed that leukaemia cells oxidise fatty acids and that they uncouple oxidative phosphorylation in order to shift ATP production from fatty acid oxidation to glycolysis. This would be in accordance with the increase in the most abundant saturated fatty acids seen in the present study, and therefore modulation of fatty acid metabolism may provide a novel strategy to treat leukaemia [27]. Through investigating the effects of lipoprotein lipase knock-down, it has been elucidated that this enzyme, a strong biomarker of CLL with previously understudied function, is involved in the regulation of fatty acid metabolism in the disease [28]. However, PUFAs are involved not only in energy metabolism, but in contributing to chemo-sensitivity in CLL [29], and therefore the different trends seen in different PUFA may be the consequences of specific signaling metabolism.

LTB4 was found to be significant owing to its apparent complete depletion in serum of CLL patients. LTB4 is in the eicosanoid family of lipids, most of which formed by the oxidation of 20-carbon essential fatty acids, that comprise prostaglandins along with prostacyclins, thromboxanes and endocannabinoids in addition to leukotrienes. Methods for profiling bioactive lipids such as eicosanoids in CLL cells to better understand the signalling cascade has been previously performed [30]. Leukotrienes are biosynthesised from arachidonic acid *via* 5-lipoxygenase (5-LO) in the body by myeloid cells and B lymphocytes [31]. Despite the abundant expression of 5-LO in B-CLL cells, LTB4 biosynthesis is believed not to occur in low differentiated malignant B lymphocytes [32]. In a recent study based on a model system imitating T-cell dependent activation of B cells, the function of the 5-LO pathway in B-CLL cells was investigated. It was revealed that under certain conditions, B-CLL cells have equal capacity to myeloid cells to biosynthesise and release LTB4. Moreover, it was proposed that LTB4 is involved in B-CLL cell activation and that leukotriene biosynthesis inhibitors similar to those employed in the treatment of asthma could be applicable in the treatment of CLL [33]. In another study, it was revealed that while intracellular concentrations of LTB4 can be higher in B-CLL cells relative to their normal counterpart, plasma concentrations of LTB4 may not be statistically different between patients and controls, the reason being that LTB4 can be metabolised into 20-OH-LTB4 that is a non-circulating metabolite [22]. It was further suggested from this study that, at least for patients with slowly proliferating tumour cells, the balance between LTB4 synthesis and inactivation can be constant. Through inactivation or immediate metabolism to 20-OH-LTB4, this could explain the absence of LTB4 in the serum samples from patients studied in our investigation.

Lipids are involved in many carcinogenic processes including cell dislodgement, invasion, migration, and proliferation. Other lipids that significantly differentiated patient groups in this study were (lyso)phospholipids. In a comparison between CLL and normal lymphocytes, it has been shown previously that phospholipid levels are significantly altered with the disease and that CLL can be characterised by phospholipid metabolism activation, with different phospholipids being increased and decreased with the disease [34]. The increased demand for lipids by tumour cells is reflected by the decreased levels of several lipids in blood. For example, lysoPC can be a useful marker of cancer progression for a range of cancers [35]. A decrease in LysoPC has already been observed in lung [36] and liver [37] cancer, phosphatidylinositols in pancreatic cancer [38] and for several types of lysophospholipids in B-cell malignancies (including CLL) [18]. LysoPCs, in contrast to PCs that function in membrane formation, perform membrane lysis and have been observed to be generally decreased in patients with leukaemia relative to controls [39]. Different lyso PCs in addition to oleamide and eicosatrienoic acid that were also found to be significant in our study have been previously included in a panel of biomarkers for differentiating early stage colorectal cancer patients from healthy controls that was shown to be more effective than the carcinoembryonic antigen biomarker usually utilised in diagnosis of the disease [40].

Signalling lipids including sphingolipids were also highlighted. Sphingolipids are critical bioactive lipids involved in proliferation, differentiation, apoptosis, inflammation, migration and autophagy, whose metabolism is altered in a range of diseases including but not limited to leukaemia [41]. They are also involved in lymphocyte trafficking *via* egress signals provided by spingosine-1-phosphate (S1P). With respect to leukaemia, sphingolipids are studied due to their part in the process of lymphocyte release into the circulation; leukemic clonal exit is modulated by the expression of sphingosine-1-phosphate receptor 1 (S1PR1) and levels of S1P in plasma of non-diseased subjects are normally high to instigate the migration of lymphocytes into circulation. S1PR1 is differentially expressed on CLL cells and is reduced when cells are in a tumour supportive microenvironment compared to when cells are free in circulation [42]. It has also been shown that the expression of S1P1 is reduced in CLL B cells, causing defective egress of CLL B-cells contributing to their enhanced survival [43]. When cells are in free circulation they migrate towards S1P which coincides with their increased chemosensitivity [42]. In a model targeted to reduce CLL malignancy *via* deficiency in XBP-1, the transcription factor associated to endoplasmic reticulum stress that promotes progression of CLL, increased surface expression of S1P1 was induced, rendering disadvantage to CLL cell survival [44]. S1P was significantly lower in both A and I relative to C. This is consistent with the notion that plasma levels of non-diseased subjects are normally higher than in CLL patients.

Ten metabolites were selected for further investigation through validation with an independent analytical technique, six of which were significantly increased in patients with the aggressive form of the disease relative to either indolents or controls and two of which were also able to discriminate indolents from controls. Linoleamide in addition to acylcarnitines (acetyl-, hexanoyl-, octanoyl-, decanoyl-, hexadecanoyl-) were validated as significant markers of CLL in its aggressive form relative to the indolent form or controls. As well as being markers of the aggressive form, acetylcarnitine and hexannoylcarnitine were also distinguishable markers of indolent and control subjects. This could implicate the utility of screening these compounds both to diagnose CLL (irrespective of aggressiveness) and also to potentially distinguish patients with the more aggressive form of the disease by way of stratification. Moreover, from ROC analysis, acetylcarinite, hexanoylcarnitine and octanoylcarnitine were revealed as being the best markers of CLL, owing to an increased significance when grouped together as a panel of markers. In terms of depicting biomarkers for classifying the aggressive state of CLL, grouping acetylcarnitines was promising; however this panel was surpassed by the combination of selected acylcarnitines and fatty acid amides. Abnormal expression of carnitines in patients with malignancies is well known. Furthermore, although not to the level of insufficiency, significant transient decreases in free carnitine and total carnitine in different stages of leukaemia that can affect the evolution of the disease have been previously reported [45]. Changes in carnitine levels are related to fatty acid metabolism. CLL cells have been reported to exhibit a greater dependency on peroxisome proliferator activated receptor – alpha (PPAR-α) regulated oxidation of fatty acids leading to these cells having a higher fat-burning rate than such as myocytes, that is not related to diet [46]. In CLL cells, PPAR-α is thought to aid in CLL cell survival against cytotoxic stressors including chemotherapeutic drugs as well as hypoxia and lack of nutrients [46]. PPAR-α is also closely associated to the metabolism of fatty acid amides. For example PPAR-α agonist oleoylethanolamide was observed to be significantly high in patients such that its plasma concentration was directionally related to the number of circulating leukemic cells [47]. In that study, it was suggested that oleoylethanolamide is produced in CLL cells as a lipolytic factor that later plays a role in drug resistance as well as cachexia. Another discovered therapeutic target with respect to fatty acid amides in leukaemia is fatty acid amide hydrolase (FAAH) [48]. Fatty acid amides are bioactive signalling lipids and FAAH catalyses the hydrolysis of these bioactive compounds such as oleamide and palmitoylethanolamide [49].

With the aim of elucidating potential biomarkers to be used in the screening of serum from potential CLL patients in order to improve diagnostics, prognostics and treatment perspective, metabolomics has been applied through LC-MS based technology to both fingerprint metabolomes of patients with the aggressive form or indolent form of CLL in addition to healthy volunteers and to validate a panel of biomarkers that could be used clinically. Fingerprinting revealed a host of significant differences in serum samples that could distinguish patients with the disease and also, in most cases differentiate the stage of the disease that could be crucial in improving treatment. Six of the ten metabolites used in validation were significantly increased in patients with the aggressive form of the disease relative to either indolents or controls and two of which were also able to discriminate indolents from controls. Forming a panel of selected acylcarnitines and fatty acid amides, it was possible to reach a highly specific and sensitive diagnostic approach. Although treatment may not always be the best outcome for leukaemia patients, and may even promote tumour progression [50, 51], our research offers the potential to improve diagnosis which could aid in the decision if and when treatment is in fact a viable option.

## MATERIALS AND METHODS

### Ethics statement

Patients and controls were recruited in the Department of Haematology at the Medical University in Bialystok (Poland), with the approval of the Local Ethics Committee. Written informed consent from all participants involved in the study was obtained. The study group was composed of Caucasians of European ancestry, so obtained results cannot be generalized to other ethnicities, as the effect of genetic variations on metabolism needs to be considered [52].

### Blood collection

Blood was collected from two groups of patients with newly diagnosed chronic lymphocytic leukaemia: patients with a stable state of the disease (I, “indolent”) *n* = 51, and patients with a progressive state of the disease (A, “aggressive”) *n* = 42, requiring treatment (treatment indication according to IW CLL 2008 recommendation) Detailed characteristics of patients in presented in Table S2 (found in Supplementary Information). A control group contained 45 healthy subjects (C), sex and age matched to the patient's group. In total, blood samples of 138 humans were collected.

Venous fasting blood samples were drawn into syringes containing clotting activator. Blood samples were allowed to clot and serum was obtained by centrifugation at 1300 × *g* for 30 min at 4°C. Aliquots of the serum were stored at −80°C until analysis.

### Chemicals and reagents

Ultrapure water, used to prepare all the aqueous solutions was obtained “in-house” from a Milli-Qplus185 system (Millipore, Billerica, MA, USA). Standards used for confirmation of metabolite identity in the fingerprinting study as well as LC-MS grade acetonitrile and analytical grade formic acid were purchased from Fluka Analytical (Sigma-Aldrich Chemie GmbH, Steinheim, Germany). Deuterated (D3) standards of acylcarnitines (acetylcarnitine, butyrylcarnitine, hexanoylcarnitine, octanoylcarnitine, decanoylcarnitine and palmitoylcarnitine) used in validation study were purchased from Chromsystems (Munich, Germany).

### Metabolic fingerprinting with ESI-QTOF-MS

### Method

Metabolic fingerprinting of serum samples was performed with LC-QTOF-MS (6520, Agilent Technologies) method previously applied to study serum of patients with B-cell malignancies [18]. Detailed description of this methodology can be found in Supplementary Information. To control system's stability and performance [53] and the reproducibility of the sample treatment procedure QC samples were prepared by pooling equal volumes of serum from each investigated sample. Seven QC samples were prepared independently of this pooled serum, following the same procedure as for the rest of samples. Each QC sample was injected only once, QCs were injected at the beginning of the run and after every 8th sample.

### Data treatment

The resulting data file was cleaned of background noise and unrelated ions by the Molecular Feature Extraction (MFE) tool in Mass Hunter Qualitative Analysis Software B.04.00 (Agilent). The MFE algorithm uses the accuracy of mass measurements to group ions related by charge-state envelope, isotopic distribution and/or the presence of adducts and dimmers. The MFE parameters were the same as described previously [18]. Briefly, the limit for the background noise was set to 200 counts, and to find coeluting adducts of the same feature, the following adduct settings were applied: +H, +Na, +K in positive ionization, and: −H, +HCOO in negative ionization. Dehydratation neutral losses were also allowed. The MFE then created a list of all metabolic features described by accurate mass, retention time and abundance. Exact mass databases quoted below were then searched for hits to identify compounds.

Data treatment was performed according to current standards for the treatment of LC-MS metabolomics data [53]. Alignment, filtering and statistical analysis were performed with Mass Profiler Professional 2.2 (Agilent) software. Parameters applied for the alignment were 1% for retention time correction and 20 ppm for correction of the mass. Differences between serum metabolites in all comparisons (C *vs* A, C *vs* I, and A *vs* I) were evaluated for individual metabolites by means of a Welch's *t*-test assuming unequal variance. *p*-values were calculated for the data transformed by applying log (base 2) for intensities in order to approximate a normal distribution. Multiple testing correction was performed by use of Benjamini Hochberg FDR test. The normality of distribution was assessed using the Shapiro-Wilk test performed with STATISTICA 9.1 (StatSoft) software. Accurate masses of statistically significant features were searched against the METLIN, KEGG, LIPIDMAPS and HMDB databases. SIMCA-P+ 12.0 (Umetrics) was used for multivariate statistical analysis.

### Compound identification

The identities of compounds that were found to be significant for class separation (Tables 1–3) were confirmed by LC-MS/MS analysis as described previously [18]. Confirmation with standards was performed by comparing retention time, isotopic distribution and fragments of commercially (Sigma–Aldrich Chemie GmbH, Steinheim, Germany) available reagents with those obtained in real samples.

### Validation of selected metabolites by LC-QQQ-MS

### Method

To analyse the samples a new methodology was developed, based on reversed-phase UHPLC (1290 Infinity, Agilent Technologies) coupled to an ESI(AJS)-QQQ-MS (6460, Agilent Technologies) mass spectrometer. During the method development, standards of acylcarnitines (acetylcarnitine, butyrylcarnitine, hexanoylcarnitine, octanoylcarnitine, decanoylcarnitine, palmitoylcarnitine) and fatty acid amides (dodecanamide, hexadecanamide, oleamide, linoleamide) were used. Detailed description of developed method can be found in Supplementary Information. Analyses were controlled with QC samples consisted of equal volumes of serum from each investigated sample. QCs were prepared following the same procedure as the rest of samples for QQQ analysis and were injected after every 10th sample.

### Data treatment

Acylcarnitines were quantitated according to the response factor of the respective internal standard. Labelled fatty acid amides were not commercially available, therefore the amount of free fatty acid amides (FFAAs) in the samples were expressed as areas under the respective peaks. For data generated by LC-QQQ, normality of distribution was tested by the Shapiro-Wilk test, and depending on normality, Welch's *t*-test or Mann Whitney *U*-test were used to evaluate differences for the comparisons of C *vs* A, C *vs* I, and A *vs* I.

Data filtering and statistical analysis of fingerprinting data was performed with Mass Profiler Professional 2.2 (Agilent). The Shapiro-Wilk test and statistical analysis of validation data and receiver operating characteristic (ROC) curves were performed with MATLAB 7.10 R2010a (MathWorks Inc., Natick, MA, USA).

## SUPPLEMENTARY MATERIALS TABLES


