# Supplementary material for: To treat or not to treat: metabolomics reveals biomarkers for treatment indication in chronic lymphocytic leukaemia patients
Source: Oncotarget. 2016 Mar 14;7(16):22324–38. doi: 10.18632/oncotarget.8078 (PMC5008363; doi:10.18632/oncotarget.8078)
Supplement: Supplementary file 1 [file oncotarget-07-22324-s001.pdf]

To treat or not to treat: metabolomics reveals biomarkers for treatment indication in chronic lymphocytic leukaemia patients

Supplementary Materials

Supplementary Table S1: Clinical features of active disease

1.	Evidence of progressive marrow failure as manifested by the development or worsening of anemia and/or thrombocytopenia.
2.	Massive (i.e. at least 6 cm below the left costal margin) or progressive or symptomatic splenomegaly.
3.	Massive nodes (i.e. at least 10 cm in the longest diameter) or progressive or symptomatic lymphadenopathy.
4.	Progressive lymphocytosis with an increase of more than 50% over a 2-month period or lymphocyte doubling time (LDT) of less than 6 months. LDT can be obtained by linear regression extrapolation of absolute lymphocyte counts obtained at intervals of 2 weeks over an observation period of 2 to 3 months. In patients with initial blood lymphocyte counts of less than 30×10^9/L (30 000/μL), LDT should not be used as a single parameter to define treatment indication. In addition, factors contributing to lymphocytosis or lymphadenopathy other than CLL (eg, infections) should be excluded.
5.	Autoimmune anaemia and/or thrombocytopenia that is poorly responsive to corticosteroids or another standard therapy.
6.	Constitutional symptoms defined as any one or more of the following disease-related symptoms or signs:<br>a. Unintentional weight loss of 10% or more within the previous 6 months;<br>b. significant fatigue (i.e. ECOG PS 2 or worse; inability to work or perform usual activities);<br>c. fevers higher than 100.5°F or 38.0°C for 2 or more weeks without evidence of infection;<br>d. night sweats for more than 1 month without evidence of infection.

Supplementary Table S2: Clinical and molecular characteristics of studied patients

Number of patients	93
Median of age (range)	65 (40–85)
Sex:<br>Female<br>Male	 48<br>45
Rai stage, number of patients<br>0<br>I<br>II<br>III<br>IV	 10 (10,8%)<br>19 (20,4%)<br>37 (39,8%)<br>19 (20,4%)<br>8 (8,6%)

Hierarchical cytogenetics subgroup	
Sole 13q deletion	30.1%
Normal	34%
Trisomy 12 (no 17p13 or 11q22 deletion)	10.7%
11q22 deletion (no 17p13 deletion)	14.3%
17p13 deletion	10,7%
ZAP70 > 30%	30.4%
Patients at “wait and watch” strategy (stable state of the disease)	51
Patients qualified to treatment (progressive state of the disease)	42

HGB – haemoglobin; β2m - beta-2-microglobulin; LDH - lactate dehydrogenase; TB - trephine biopsy; BM – bone marrow; PLT –platelets count; WBC- white blood cells; CR-complete remission; PR-partial remission; SD-stable disease.

Metabolic fingerprinting with ESI-QTOF-MS

Serum samples were prepared as follows. Protein precipitation and metabolite extraction was performed by adding 1 volume of serum to 3 volumes of cold (−20°C) mixture of methanol and ethanol (1:1). Samples were then vortex-mixed and stored at −20°C for 5 min. The pellet was removed by centrifuging at 16 000 × g for 20 min at 4°C, and the supernatant was filtered through a 0.22 µm nylon filter. Analyses were performed with the HPLC system consisting of a degasser, two binary pumps, and thermostated autosampler, maintained at 4°C (1200 series, Agilent); 10 µL of extracted serum sample was applied to a reversed-phase column (Discovery HS C_{18} 15 cm × 2.1 mm, 3 µm; Supelco) with a guard column (Discovery HS C_{18} 2 cm × 2.1 mm, 3 µm; Supelco) thermostated at 40°C. The system was operated at a flow rate 0.6 mL/min with solvent A composed of water with 0.1% formic acid, and solvent B composed of acetonitrile with 0.1% formic acid. The gradient started from 25% B to 95% B in 35 min, and returned to starting conditions in 1 min, keeping the reequilibration at 25% B for 9 min. Data were collected in positive and negative ESI ion modes in separate runs on a QTOF (Agilent 6520) operated in full scan mode from 50 to 1000 *m*/*z* for positive, and 50 to 1050 *m*/*z* for negative ion mode with a scan rate of 1 scan per second. Accurate mass measurements were obtained by means of an automated calibrant delivery system using a dual-nebulizer ESI source that continuously introduces a calibrating solution, which contains reference masses at *m/z* 121.0509 ($C_5H_4N_4$) and 922.0098 ($C_{18}H_{18}O_6N_3P_3F_{24}$) for positive and 112.9856 ($C_2O_2F_3(NH_4)$) and 1033.9881 ($C_{18}H_{18}O_6N_3P_3F_{24}$) for negative ionization mode. The capillary voltage was set to 3000 V for positive and 4000 V for negative ionization mode; the nebulizer gas flow rate was 10.5 L/min. Samples were analyzed in randomized order in two runs (first for positive and second for negative ion mode). At the beginning of each run, a batch of 5 injections of a QC sample was used to condition the column.

Validation of selected metabolites by LC-QQQ-MS

Sample preparation

To 25 µL of each serum sample, 50 µL of deuterated IS mixture was added. Subsequently 200 µL of cold ethanol: methanol (1:1, v/v) was added to achieve protein precipitation. Samples were vortex-mixed for 1 min, incubated on ice for 5 min and centrifuged at 15,400 × g for 20 min at 4°C. 180 µL of the supernatant were drawn and transferred to a vial with glass insert, which was kept in the autosampler at 15°C until injection.

LC-QQQ-MS settings

In the final method, 2 µL of sample was injected onto a reversed-phase column (Zorbax Eclipse Plus C8; 150 × 2.1 mm, 1.8 µm; Agilent Technologies) thermostated at 55°C and compounds were gradient-eluted at 0.6 mL/min, in 4.5 min. Solvent A of the mobile phase was water with 0.1% formic acid and solvent B acetonitrile with 0.1% formic acid. The gradient started from 4% B and was kept for 0.25 min, then increased to 86% B in 2.05 min, and this percentage was maintained for 1 min. The % B was further increased to 97% in 0.1 min. The system was allowed to return to initial conditions after 0.1 min to re-equilibrate for 0.9 min before starting the new injection. MS parameters were as follows: nebulizer gas flow rate 8 L/min, source temperature 200°C, nebulizer pressure 60 psi, sheath gas flow 8 L/min and sheath gas temperature 250°C. Capillary and Nozzle voltages were 4000 V and at 2000 V respectively. The analyses were carried out in positive ion mode. The system was operated

in dynamic Multiple Reaction Monitoring (MRM) mode, in which the precursor ion of each compound was isolated by the first quadrupole, fragmented in the collision cell and the most characteristic fragments were selected by the third quadrupole. Specific transitions (parent/product ion pairs) were acquired for each compound, which allowed for their definitive identification and quantification. Each transition was pre-optimised adjusting the collision energy, fragmentor voltage and product ion mass-to-charge ratio. Final parameters are displayed in Table S3.

Supplementary Table S3: Dynamic MRM transitions

Compound Name	Precursor Ion (m/z)	Product Ion (m/z)	Fragmentor (V)	Collision Energy (V)	Cell Accelerator Voltage (V)	RT (min)
Acetylcarnitine	204.1	85.0	146	14	7	0.71
Acetylcarnitine-D3	207.1	85.0	80	15	7	0.71
Butyrylcarnitine	232.2	85.0	128	14	7	1.52
Butyrylcarnitine-D3	235.2	99.0	110	19	7	1.52
Hexanoylcarnitine	260.2	85.0	146	14	7	1.85
Hexanoylcarnitine –D3	263.2	85.0	110	19	7	1.85
Octanoylcarnitine	288.2	85.0	140	14	7	2.1
Octanoylcarnitine -D3	291.2	85.0	110	15	7	2.1
Decanoylcarnitine	316.2	85.0	146	18	7	2.3
Decanoylcarnitine -D3	319.3	85.0	80	19	7	2.3
Palmitoycarnitine	400.3	85.0	176	22	7	2.8
Palmitoycarnitine -D3	403.4	85.0	110	27	7	2.8
Dodecanamide	200.2	57.1	146	18	7	3
Hexadecanamide	256.3	57.1	122	18	7	3.66
Oleamide	282.3	69.1	152	22	7	3.8
Linoleamide	280.3	55.1	134	34	7	3.5
